# Supplementary material for: Deletion of wfs1 Impairs Oligodendrocyte Precursor Cells Dorsal Distribution and Myelination Through the wfs1-hmgcs1 Axis in Zebrafish
Source: Biology (Basel). 2026 Aug 16;15(16):1402. doi: 10.3390/biology15161402 (PMC13510151; doi:10.3390/biology15161402)
Supplement: Supplementary file 1 [file biology-15-01402-s001.zip › File S1-Transcriptome sequencing data and differentially expressed genes (DEGs) in wfs1-- zebrafish at 5 dpf/2.KEGG/wfs1KOvsWT/GSEA/wfs1KO/GLYCEROLIPID_METABOLISM/GLYCEROLIPID_METABOLISM(DRE00561).html]

Details for gene set GLYCEROLIPID\_METABOLISM(DRE00561)[GSEA]

|  || Dataset | deg.sample |
| Phenotype | sample.cls |
| Upregulated in class | wfs1KO |
| GeneSet | GLYCEROLIPID\_METABOLISM(DRE00561) |
| Enrichment Score (ES) | 0.32302547 |
| Normalized Enrichment Score (NES) | 1.37089 |
| Nominal p-value | 0.014736842 |
| FDR q-value | 0.24656262 |
| FWER p-Value | 0.906 |
Table: GSEA Results Summary

  

Fig 1: Enrichment plot: GLYCEROLIPID\_METABOLISM(DRE00561)      
 Profile of the Running ES Score & Positions of GeneSet Members on the Rank Ordered List

  

| PROBE | DESCRIPTION (from dataset) | GENE SYMBOL | GENE\_TITLE | RANK IN GENE LIST | RANK METRIC SCORE | RUNNING ES | CORE ENRICHMENT || 1 | 724010\_1 | dgkaa |  |  | 12 | 4.962 | 0.0794 | Yes |
| 2 | 565117 | cel.2 |  |  | 426 | 3.195 | 0.1179 | Yes |
| 3 | 450083 | gla |  |  | 814 | 2.368 | 0.1438 | Yes |
| 4 | 322481 | cel.1 |  |  | 1043 | 2.053 | 0.1697 | Yes |
| 5 | 368239 | aldh2.2 |  |  | 1221 | 1.881 | 0.1943 | Yes |
| 6 | 799099\_1 | lpin1 |  |  | 1367 | 1.749 | 0.2179 | Yes |
| 7 | 445326\_1 | akr1a1a |  |  | 1428 | 1.705 | 0.2435 | Yes |
| 8 | 572841 | mboat1 |  |  | 1662 | 1.561 | 0.2613 | Yes |
| 9 | 447920 | aldh3a2b |  |  | 1830 | 1.444 | 0.2792 | Yes |
| 10 | 559124 | ppap2d |  |  | 2971 | 1.043 | 0.2602 | Yes |
| 11 | 334270 | agk |  |  | 3184 | 0.996 | 0.2695 | Yes |
| 12 | 565122 | si:dkey-172j4.3 |  |  | 3472 | 0.936 | 0.2756 | Yes |
| 13 | 557324 | dgkb |  |  | 3678 | 0.897 | 0.2835 | Yes |
| 14 | 393997 | lipca |  |  | 4285 | 0.778 | 0.2770 | Yes |
| 15 | 793926 | tkfc |  |  | 4368 | 0.766 | 0.2868 | Yes |
| 16 | 564739 | LOC564739 |  |  | 4494 | 0.746 | 0.2948 | Yes |
| 17 | 565316 | dgat2 |  |  | 4579 | 0.736 | 0.3040 | Yes |
| 18 | 567728\_1 | LOC567728 |  |  | 5105 | 0.666 | 0.2982 | Yes |
| 19 | 100005587 | aldh9a1a.1 |  |  | 5134 | 0.663 | 0.3080 | Yes |
| 20 | 559124\_1 | ppap2d |  |  | 5172 | 0.659 | 0.3175 | Yes |
| 21 | 567414\_2 | agpat9l |  |  | 5326 | 0.645 | 0.3230 | Yes |
| 22 | 560638 | si:ch73-21k16.1 |  |  | 5961 | 0.591 | 0.3126 | No |
| 23 | 110438373 | LOC110438373 |  |  | 6078 | 0.579 | 0.3183 | No |
| 24 | 563806 | plpp2a |  |  | 6278 | 0.559 | 0.3210 | No |
| 25 | 399481 | aldh9a1b |  |  | 6718 | 0.518 | 0.3155 | No |
| 26 | 558422 | lipin2 |  |  | 7174 | 0.475 | 0.3089 | No |
| 27 | 558437 | gpat2 |  |  | 7304 | 0.466 | 0.3123 | No |
| 28 | 335485 | plpp2b |  |  | 8056 | 0.419 | 0.2954 | No |
| 29 | 393462 | aldh2.1 |  |  | 8615 | 0.394 | 0.2842 | No |
| 30 | 100000026 | aldh3a1 |  |  | 8825 | 0.379 | 0.2838 | No |
| 31 | 450063 | mogat2 |  |  | 8878 | 0.376 | 0.2882 | No |
| 32 | 101884800 | LOC101884800 |  |  | 9188 | 0.353 | 0.2841 | No |
| 33 | 557680 | plpp3 |  |  | 9279 | 0.348 | 0.2869 | No |
| 34 | 692278 | mboat2b |  |  | 10139 | 0.294 | 0.2646 | No |
| 35 | 101887155 | pnpla2 |  |  | 10760 | 0.257 | 0.2493 | No |
| 36 | 100137124 | dgkh |  |  | 11010 | 0.243 | 0.2454 | No |
| 37 | 436958\_1 | gpat3 |  |  | 11354 | 0.224 | 0.2382 | No |
| 38 | 641489 | zgc:123305 |  |  | 11735 | 0.204 | 0.2295 | No |
| 39 | 406734 | agpat3 |  |  | 11853 | 0.198 | 0.2290 | No |
| 40 | 100148835 | dgkzb |  |  | 12306 | 0.174 | 0.2176 | No |
| 41 | 436610 | pnpla3 |  |  | 12510 | 0.164 | 0.2139 | No |
| 42 | 100332911 | gpam |  |  | 13236 | 0.129 | 0.1931 | No |
| 43 | 406265 | agpat4 |  |  | 13450 | 0.118 | 0.1883 | No |
| 44 | 393096 | lipg |  |  | 13509 | 0.115 | 0.1884 | No |
| 45 | 431758 | plpp5 |  |  | 13667 | 0.108 | 0.1852 | No |
| 46 | 494083 | mogat3b |  |  | 14136 | 0.085 | 0.1718 | No |
| 47 | 492710 | aldh16a1 |  |  | 14167 | 0.084 | 0.1722 | No |
| 48 | 777624 | agpat2 |  |  | 14299 | 0.078 | 0.1694 | No |
| 49 | 378960 | mgll |  |  | 14379 | 0.075 | 0.1681 | No |
| 50 | 553452 | si:dkey-180p18.9 |  |  | 14479 | 0.070 | 0.1661 | No |
| 51 | 553380 | dgkza |  |  | 14928 | 0.050 | 0.1528 | No |
| 52 | 799805\_1 | akr1a1b |  |  | 15001 | 0.046 | 0.1513 | No |
| 53 | 794598 | LOC794598 |  |  | 15191 | 0.038 | 0.1460 | No |
| 54 | 100150463\_1 | dgke |  |  | 15224 | 0.036 | 0.1455 | No |
| 55 | 567728 | LOC567728 |  |  | 15292 | 0.033 | 0.1440 | No |
| 56 | 100331649 | dgki |  |  | 16424 | -0.015 | 0.1086 | No |
| 57 | 100334794 | plpp4 |  |  | 16965 | -0.033 | 0.0922 | No |
| 58 | 570678 | glyctk |  |  | 17707 | -0.063 | 0.0699 | No |
| 59 | 678522 | gpat4 |  |  | 17897 | -0.071 | 0.0651 | No |
| 60 | 30354 | lpl |  |  | 18171 | -0.084 | 0.0579 | No |
| 61 | 567414\_1 | agpat9l |  |  | 18220 | -0.086 | 0.0577 | No |
| 62 | 325875 | dgat1a |  |  | 18907 | -0.118 | 0.0381 | No |
| 63 | 100136866 | zgc:172295 |  |  | 19211 | -0.131 | 0.0307 | No |
| 64 | 564613 | abhd16a |  |  | 19349 | -0.137 | 0.0286 | No |
| 65 | 570695 | mboat2a |  |  | 19401 | -0.139 | 0.0292 | No |
| 66 | 100142647 | mogat3a |  |  | 19741 | -0.155 | 0.0210 | No |
| 67 | 100536469 | LOC100536469 |  |  | 20155 | -0.173 | 0.0108 | No |
| 68 | 334197 | aldh7a1 |  |  | 21094 | -0.221 | -0.0151 | No |
| 69 | 569053 | plpp1a |  |  | 21219 | -0.226 | -0.0154 | No |
| 70 | 100334707 | LOC100334707 |  |  | 22479 | -0.295 | -0.0502 | No |
| 71 | 406554 | lclat1 |  |  | 22664 | -0.305 | -0.0511 | No |
| 72 | 100006238 | aldh9a1a.2 |  |  | 23161 | -0.338 | -0.0613 | No |
| 73 | 436958 | gpat3 |  |  | 23225 | -0.343 | -0.0577 | No |
| 74 | 450083\_1 | gla |  |  | 23329 | -0.350 | -0.0553 | No |
| 75 | 767778 | agpat5 |  |  | 24278 | -0.412 | -0.0785 | No |
| 76 | 799805 | akr1a1b |  |  | 25680 | -0.501 | -0.1145 | No |
| 77 | 436731 | dgat1b |  |  | 27949 | -0.736 | -0.1740 | No |
| 78 | 323653 | aldh3a2a |  |  | 28024 | -0.745 | -0.1643 | No |
| 79 | 334270\_1 | agk |  |  | 28037 | -0.747 | -0.1527 | No |
| 80 | 799099 | lpin1 |  |  | 28128 | -0.760 | -0.1433 | No |
| 81 | 100150463\_2 | dgke |  |  | 28377 | -0.800 | -0.1382 | No |
| 82 | 572841\_1 | mboat1 |  |  | 29023 | -0.926 | -0.1436 | No |
| 83 | 335485\_1 | plpp2b |  |  | 29460 | -1.049 | -0.1404 | No |
| 84 | 100331214 | LOC100331214 |  |  | 29706 | -1.130 | -0.1300 | No |
| 85 | 100006238\_1 | aldh9a1a.2 |  |  | 29971 | -1.231 | -0.1185 | No |
| 86 | 445326 | akr1a1a |  |  | 30087 | -1.279 | -0.1015 | No |
| 87 | 100005587\_1 | aldh9a1a.1 |  |  | 31308 | -2.447 | -0.1005 | No |
| 88 | 100536795 | wu:fb14d09 |  |  | 31372 | -2.574 | -0.0611 | No |
| 89 | 724010 | dgkaa |  |  | 31827 | -4.806 | 0.0020 | No |
Table: GSEA details [plain text format]

  

Fig 2: GLYCEROLIPID\_METABOLISM(DRE00561)      
 Blue-Pink O' Gram in the Space of the Analyzed GeneSet

  

Fig 3: GLYCEROLIPID\_METABOLISM(DRE00561): Random ES distribution      
 Gene set null distribution of ES for **GLYCEROLIPID\_METABOLISM(DRE00561)**
